# Draft Genome Sequences of Nine Enteropathogenic *Escherichia coli* Strains from Kenya

**DOI:** 10.1128/genomeA.00582-14

**Published:** 2014-06-12

**Authors:** Tracy H. Hazen, Michael S. Humphrys, John Benjamin Ochieng, Michele Parsons, Cheryl A. Bopp, Ciara E. O’Reilly, Eric Mintz, David A. Rasko

**Affiliations:** a Institute for Genome Sciences, University of Maryland School of Medicine, Baltimore, Maryland, USA; b Kenya Medical Research Institute/Centers for Disease Control and Prevention, Kisumu, Kenya; c Division of Foodborne, Waterborne, and Environmental Diseases, National Center for Emerging and Zoonotic Infectious Diseases, Centers for Disease Control and Prevention, Atlanta, Georgia, USA; d Department of Microbiology and Immunology, University of Maryland School of Medicine, Baltimore, Maryland, USA

## Abstract

We report here the draft genome sequences of nine enteropathogenic *Escherichia coli* (EPEC) strains isolated from children in Kenya who died during hospitalization with diarrhea. Each of the isolates possess the EPEC adherence factor (EAF) plasmid encoding the bundle-forming pilus, which is characteristic of EPEC. These isolates represent diverse serogroups and EPEC phylogenomic lineages.

## GENOME ANNOUNCEMENT

Attaching and effacing *Escherichia coli* (AEEC) is characterized by the presence of the locus of enterocyte effacement (LEE) pathogenicity region that encodes a type III secretion system involved in pathogenesis (1–3). AEEC is further identified as either LEE-positive Shiga toxin-producing *E. coli* (STEC) or enteropathogenic *E. coli* (EPEC). EPEC strains contain the LEE region but not the Shiga toxin-encoding phage, and they are considered typical EPEC when they possess the bundle-forming pilus (BFP) encoded by the EPEC adherence factor (EAF) plasmid (1, 4, 5). In contrast, LEE-positive *stx*-negative *bfp*-negative *E. coli* strains are considered atypical EPEC (4). The nine *E. coli* isolates described here are LEE positive, *stx* negative, and *bfp* positive and are therefore classified as typical EPEC based on their virulence factor content.

The genomic DNA for sequencing was isolated from an overnight culture of each isolate using the Sigma GenElute kit (Sigma-Aldrich). Genome sequencing was performed at the University of Maryland School of Medicine, Institute for Genome Sciences, at the Genome Resource Center. The genome sequences were generated using paired-end libraries with 300-bp inserts on the Illumina HiSeq 2000. The Illumina reads generated for each isolate were assembled *de novo* using the Velvet assembly program (6), with *k*-mer values determined using VelvetOptimiser version 2.1.4 (http://bioinformatics.net.au/software.velvetoptimiser.shtml). The genome assemblies contained an average of 181 contigs per isolate (range, 117 to 283), with an average G+C content of 50.4% (range, 50.3 to 50.6%) and an average genome size of 5.2 Mb (range, 5.0 to 5.4 Mb).

These nine isolates were isolated from children in Kenya who died during hospitalization with diarrhea (7). These EPEC isolates have diverse serogroups (O126, O119, O111, O53, O127, O55, and O114). Phylogenomic analysis of the whole genomes demonstrated that these isolates occur in two *E. coli* phylogroups (B1 and B2) (8, 9) and occupy four EPEC phylogenomic lineages. Among the four EPEC isolates in phylogroup B2 was isolate S6995 (O127), which was identified in the EPEC1 phylogenomic lineage, with the most widely studied prototype EPEC isolate E2348/69 (10). Also from phylogroup B2 was EPEC isolate S6400 (O119), which was identified in the recently described EPEC4 phylogenomic lineage (11). The remaining two EPEC isolates identified in phylogroup B2, S6966 (O53) and S7438 (O55), were present in unassigned phylogenomic lineages. The five EPEC isolates identified in phylogroup B1, S7005 (O126), S6685 (O114), S5274 (O111), S7380 (serogroup unknown), and S6662 (O111), were all present in the EPEC2 phylogenomic lineage, which contains the EPEC prototype isolate B171 (12).

The genome sequencing of these nine EPEC isolates demonstrates that the EPEC isolates associated with diarrheal illness of children in Kenya exhibit considerable genomic diversity that was not previously appreciated.

### Nucleotide sequence accession numbers.

The genome assemblies have been deposited at DDBJ/EMBL/GenBank with accession no. JICI00000000, JICH00000000, JICG00000000, JICF00000000, JICE00000000, JICD00000000, JICC00000000, JICB00000000, and JICA00000000, for isolates S6966, S6995, S7005, S7438, S6400, S7380, S6662, S5274, and S6685, respectively.
